# A commercial arbuscular mycorrhizal inoculum increases root colonization across wheat cultivars but does not increase assimilation of mycorrhiza‐acquired nutrients

**DOI:** 10.1002/ppp3.10094

**Published:** 2020-01-03

**Authors:** Ashleigh J. Elliott, Tim J. Daniell, Duncan D. Cameron, Katie J. Field

**Affiliations:** ^1^ Centre for Plant Sciences School of Biology Faculty of Biological Sciences University of Leeds Leeds UK; ^2^ Department of Animal and Plant Sciences University of Sheffield Sheffield UK

**Keywords:** arbuscular mycorrhiza, carbon, inoculant, nitrogen, phosphorus, sustainable agriculture, symbiosis, wheat

## Abstract

Production and heavy application of chemical‐based fertilizers to maintain crop yields is unsustainable due to pollution from run‐off, high CO_2_ emissions, and diminishing yield returns. Access to fertilizers will be limited in the future due to rising energy costs and dwindling rock phosphate resources. A growing number of companies produce and sell arbuscular mycorrhizal fungal (AMF) inoculants, intended to help reduce fertilizer usage by facilitating crop nutrient uptake through arbuscular mycorrhizas. However, their success has been variable. Here, we present information about the efficacy of a commercially available AMF inoculant in increasing AMF root colonization and fungal contribution to plant nutrient uptake, which are critical considerations within the growing AMF inoculant industry.

**Summary**

- Arable agriculture needs sustainable solutions to reduce reliance on large inputs of nutrient fertilizers while continuing to improve crop yields. By harnessing arbuscular mycorrhizal symbiosis, there is potential to improve crop nutrient assimilation and growth without additional inputs, although the efficacy of commercially available mycorrhizal inocula in agricultural systems remains controversial.
- Using stable and radioisotope tracing, carbon‐for‐nutrient exchange between arbuscular mycorrhizal fungi and three modern cultivars of wheat was quantified in a non‐sterile, agricultural soil, with or without the addition of a commercial mycorrhizal inoculant.
- While there was no effect of inoculum addition on above‐ground plant biomass, there was increased root colonization by arbuscular mycorrhizal fungi and changes in community structure. Inoculation increased phosphorus uptake across all wheat cultivars by up to 30%, although this increase was not directly attributable to mycorrhizal fungi. Carbon‐for‐nutrient exchange between symbionts varied substantially between the wheat cultivars.
- Plant tissue phosphorus increased in inoculated plants potentially because of changes induced by inoculation in microbial community composition and/or nutrient cycling within the rhizosphere. Our data contribute to the growing consensus that mycorrhizal inoculants could play a role in sustainable food production systems of the future.

## Graphical abstract

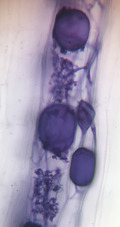

## 1 INTRODUCTION

In the last 50 years, rising food demand has driven a near‐doubling of grain yields (Food & Agriculture Organization of the United Nations, 2017). This has been achieved by the breeding of high yielding crop cultivars, development of chemical fertilizers and pesticides, and irrigation (Foley, 2005). However, agricultural fertilizer application now releases as much nitrogen (N) and phosphorus (P) into the environment as all natural processes combined (Tilman et al., 2001). In addition, depletion of the natural resources required for fertilizer manufacture (e.g., rock phosphate; Cordell, Drangert, & White, 2009), environmental damage via leaching and run‐off, and high CO_2_ emissions during production and application (Robertson & Vitousek, 2009; Goucher, Bruce, Cameron, Koh, & Horton, 2017) mean continued usage of fertilizers at current rates of application is unsustainable. As a result, future food security is far from assured, especially when considering the growing human population and changing climate. To ensure future food systems remain sustainably productive and meet United Nations Sustainable Development Goals for Zero Hunger, Life on Land and Climate Action (UN, 2015), agricultural fertilizer dependence must be reduced.

>70% of vascular plants, including most crops, form symbioses with arbuscular mycorrhizal fungi (AMF) (Brundrett & Tedersoo, 2018). Through extensive networks of extra‐radical mycelium (ERM), AMF access soil mineral nutrients inaccessible to their host plant, including nutrient pools beyond the root depletion zone (Smith & Read, 2008) and may also mineralize organic P sources (Koide & Kabir, 2000). However, it is possible that outside of axenic systems, organic nutrient mineralization by AMF may be limited in relative importance, or driven by other soil microorganisms (Joner, Aarle, & Vosatka, 2000). There is a general consensus that arbuscular mycorrhizas (AMs) contribute to N uptake although the amount may be quantitatively insignificant, at least in comparison to P acquisition (Smith & Read, 2008), particularly in agricultural systems with inorganic N applications, although this may be attributable to other symbiotic soil fungi (Hoysted et al., 2019). AMs may be more beneficial for plant P uptake because inorganic N sources are far more mobile in the soil than P and consequently do not become depleted in the rhizosphere, meaning root access is less limited (Smith & Smith, 2011). Plant‐available P generally occurs as negatively charged orthophosphate (Smith, Jakobsen, Gronlund, & Smith, 2011) which is highly reactive with aluminum, iron and calcium, forming inorganic structures with low solubility and thus, mobility. AMF‐acquired nutrients are exchanged with the host plant for photosynthetically fixed carbon (C) compounds such as sugars and lipids (Helber et al., 2011; Keymer et al., 2017). There is growing interest in application of AMF within sustainable food production systems (Rillig et al., 2016; Igiehon & Babalola, 2017; Thirkell, Charters, Elliott, Sait, & Field, 2017) to help increase crop nutrient capture, thereby reducing the need for excessive application of chemical fertilizers (Püschel et al., 2017).

AMF diversity in agricultural soils has been decimated by conventional agricultural practices (Helgason, Daniell, Husband, Fitter, & Young, 1998), including overuse of fertilizers, tillage, long fallow periods, and crop rotations. These practices disturb extra‐radical hyphal network development, creating long periods where AMF have limited access to host plants and thus, a C supply (Helgason et al., 1998; Daniell, Husband, Fitter, & Young, 2001; Jansa et al., 2002). AMF diversity and abundance in agricultural soils could be improved by changing management practices which promote native AMF abundance, or by reintroducing AM fungal spores and propagules through inoculation (Lekberg & Koide, 2005).

Commercial AMF products are readily available and targeted at the general public and agricultural industry (Vosátka, Látr, Gianinazzi, & Albrechtová, 2012; Igiehon & Babalola, 2017). Inoculants aim to boost AMF spore density in soils and act as “biofertilizers,” with the intention of promoting the effective use of existing soil nutrient pools by crops (Vosátka et al., 2012; Faye et al., 2013). In order for inoculants to become widely integrated into sustainable agricultural practices, they must prove to be effective both in increasing root colonization and nutrient capture. Critically, inoculum application must compete with chemical fertilizers in terms of both cost and by improving crop yields, ensuring financial viability for farmers. While it has been shown that AMF inoculants can be applied to, and increase yields in, agricultural fields in a commercially viable way (Ceballos et al., 2013; Hijri, 2016; Zhang, Feng, & Declerck, 2018), crop yield improvements following inoculation remain unreliable, with yield reductions reported in 14.6% of trials (Hijri, 2016). This suggests AMF inocula may not be advantageous in all agricultural scenarios and case‐by‐case assessment is required (Ceballos et al., 2013; Hijri, 2016); where inoculation takes place, changing management practices will also be essential.

Plant responses to AMF colonization can vary dramatically between and within species (Klironomos, 2003; Hoeksema et al., 2010), ranging from increased plant nutrient assimilation and growth, to neutral or negative responses where fungal partners offer little or no measurable benefit (Ellouze et al., 2016; Sawers et al., 2017; Watts‐Williams, Cavagnaro, & Tyerman, 2019). There is also considerable variation between AMF species and genotypes (Munkvold, Kjøller, Vestberg, Rosendahl, & Jakobsen, 2004; Angelard, Colard, Niculita‐Hirzel, Croll, & Sanders, 2010) in tolerance to agriculturally relevant environmental variables, such as disturbance (Schnoor, Lekberg, Rosendahl, & Olsson, 2011) and high soil nutrient concentrations (Oehl et al., 2010). As such, it is possible AMF within a commercial inoculum could fail to establish (Vosátka et al., 2012) if they are not compatible with the host plants, environment, cannot compete with the soil's native AMF communities, or cannot survive standard agronomic practices (Berruti, Lumini, Balestrini, & Bianciotto, 2016). Despite this, field experiments suggest AMF from inocula can survive and persist in plant roots for at least 2 years after introduction and can occur alongside plant productivity increases (Pellegrino et al., 2012). Conflicting datasets and data shortages from field trials or field‐relevant pot‐based experiments have resulted in no clear consensus on the application of AMF inocula in sustainable food systems, underlining the urgent need for more detailed investigations (Ryan & Graham, 2018; Rillig et al., 2019).

Using a commercially available AMF inoculum (*Rhizophagus irregularis*) and non‐sterile, agricultural field soil, we addressed the following questions: (a) Does commercial AMF inoculum application result in greater wheat (*Triticum aestivum* L.) root colonization and alter the AMF community structure? (b) Does inoculation with commercial AMF result in greater fungal‐acquired nutrient gain (with correspondingly greater C allocation to fungal symbionts) and crop growth than native AMF populations within agricultural field soil? (c) Are there any differences in response to AMF inoculation between wheat cultivars? We hypothesized that applying a commercially available inoculum containing a generalist AMF species to agricultural field soil will result in greater root colonization and increased fungal‐acquired nutrient assimilation by host plants. We expect inoculated plants to allocate more plant‐fixed C to their AMF symbionts, thereby mycorrhizal nutrient gains will be offset by a greater C cost to the plants hosting a larger AMF population. Based on previous research showing cultivar‐specific differences in mycorrhizal receptivity and function (Ellouze et al., 2016; Sawers et al., 2017; Thirkell, Pastok, & Field, 2019; Watts‐Williams et al., 2019), we expect differences between the cultivars tested here in the degree of AM colonization, AM‐mediated nutrient assimilation and C allocation to AMF.

## 2 MATERIALS AND METHODS

### 2.1 Biological material and growth conditions

Wheat (*Triticum aestivum* L.—cvs: Skyfall, Avalon and Cadenza) was grown in an agricultural soil (Leeds University Farm, Tadcaster, UK) and sand mixture (1:1) in 1.1 L pots and inoculated with either a commercially available *R. irregularis* inoculum (20 g per pot) (PlantWorks Limited, Kent; *n* = 12), or a control inoculum, sterilized by autoclaving (*n* = 12). Analysis of the soil characteristics showed the soil had a pH of 7.5, soil organic C content represented ~ 2% of soil dry weight and soil solute concentrations of PO_4_^‐^, NO_3_^‐^, and NH_4_^+^ were 0.08 mg/L, 6 mg/L, and 0.04 mg/L, respectively (Holden et al., 2019). Plants were grown for 8 weeks, in glasshouse conditions (see SI). Two plastic cores, 20 mm diameter and 100 mm long, with 35 µm pore nylon mesh windows (Figure S1), were inserted into each pot (Figure S2). A 1 mm internal diameter perforated capillary tube (Portex, Smiths Medical) was installed centrally in each core. A third core was filled with glass wool and sealed with a SubaSeal (Sigma‐Aldrich) to allow below‐ground soil respiration sampling throughout the ^14^C labeling period (Field et al., 2012). Plants were harvested immediately after completion of ^33^P‐ and ^15^N‐for‐^14^C tracing at 9 weeks of age (Zadok growth stage GS30‐39), this stage was chosen for isotope tracing as it represented a period of rapid growth and high nutrient demand where the crops may rely more heavily on AM‐mediated nutrient uptake.

### 2.2 Quantifying ^33^P‐ and ^15^N for C exchange between wheat and fungi

Forty five days after planting, 150 μl of ^33^P‐Orthophosphate (1MBq; Hartmann Analytic, Braunschweig, Germany; Specific activity: cv. Skyfall 174.9 TBq/mmol, Cadenza 179.4 TBq/mmol, Avalon 180.6 TBq/mmol) and ^15^N ammonium chloride (1.5 mg/ml) (MP Biomedicals, Santa Ana, USA) in aqueous solution was supplied to one mesh core within each pot through the perforated capillary tube. In half of the pots (*n* = 6), labeled cores were left static, in the remaining pots fungal access to isotope tracers was removed by rotating cores every other day to sever hyphal connections between the plants and core soil (Figure S2). The rotated control treatment distinguishes between fungal contributions to plant nutrient uptake versus isotope diffusion outside the cores or other microbial nutrient cycling.

After 8 weeks, soil cores were sealed with anhydrous lanolin, and pots enclosed within airtight chambers; shoots were supplied with ^14^CO_2_ for a 16‐hr photoperiod via liberating 110 μl sodium bicarbonate [^14^C] 1 MBq (specific activity: all cultivars—2.13 GBq/mmol; Hartmann Analytic) using 2 ml of 10% lactic acid.

### 2.3 Plant harvest and tissue analysis

Upon detection of maximum below‐ground ^14^C flux (approx. 16 hr after labeling), the soil cores were removed from the pots and plant and soil materials were separated. Root subsamples were cleared with 10% KOH at 80°C for 60 min and stained with acidified ink (5% Pelikan black ink, 5% acetic acid, 90% distilled water) for 20 min (Vierheilig, Coughlan, Wyss, & Piché, 1998). Mycorrhizal colonization was assessed using the gridline‐intersect method with at least 100 intersects measured per sample (McGonigle, Miller, Evans, Fairchild, & Swan, 1990). All plant and soil materials were freeze‐dried and weighed. The AMF hyphal network within each pot was measured using a gridline‐intersect method over 50 fields of view (see Supporting Information) (Tennant, 1975).

### 2.4 PCR and T‐RFLP

Fungal DNA was extracted from freeze‐dried homogenized root material (10–20 mg), following the Plant DNeasy mini kit protocol (Qiagen). A region of the small subunit rRNA was amplified using a FAM labeled general eukaryotic forward primer NS31 (5′‐TTG GAG GGC AAG TCT GGT GCC‐3′) (Simon, Lalonde, & Bruns, 1992) and AMF‐specific reverse primer AML2 (5′‐GAA CCC AAA CAC TTT GGT TTC C‐3′) (Lee, Lee, & Young, 2008) (see Supporting Information for details). PCR products were triple‐digested with the restriction enzymes HpyCHIV, MboII, and Sau96I (New England Biolabs, Inc) (see Supporting Information for details). Genotyping was carried out on an ABI 3730 PRISM® capillary DNA analyser (Applied Biosystems). T‐RFLP data was analyzed using Genemapper software v. 5 (Applied Biosystems). The SSU sequences of AMF species commonly associated with agricultural soils were downloaded from GenBank and virtually digested with RestrictionMapper v. 3, to associate T‐RF’s with potential AMF species.

### 2.5 Mycorrhiza‐acquired ^33^P and total plant P analyses

Freeze‐dried plant material was homogenized by grinding to a fine powder (A10 basic, IKA mills, Oxford). ^33^P transfer from fungus‐to‐plant was quantified by digesting samples in concentrated H_2_SO_4_ (SI) and liquid scintillation on a Packard Tri‐carb 3100TR (Isotech). ^33^P transfer between symbionts was corrected for radioactive decay and measured using equations in Cameron, Johnson, Leake, and Read (2007; see SI for details). Total shoot and root P content was measured with the same digest solution using the molybdate blue method on a Jenway 6300 Visible Spectrophotometer (Murphy & Ripely, 1962).

### 2.6 Transfer of carbon from plant to fungus

^14^C content of plant and soil material was measured via sample oxidation (Model 307 Packard Sample Oxidiser; Isotech) and liquid scintillation (Packard Tri‐carb 3100TR, Isotech). ^14^C mass was quantified using the following equation (Cameron, Leake, & Read, 2006; see Supporting Information for details). Total C (^12^C + ^14^C) transferred from the plants to their AMF partners was calculated by quantifying the CO_2_ content mass in the labeling chamber and the proportion of the supplied ^14^CO_2_ which was fixed by the plants using equations from Cameron, Johnson, Read, and Leake (2008). The difference in total C between the static and rotated core represents plant‐to‐fungus C transfer.

### 2.7 ^15^N tissue analysis

To quantify fungus‐to‐plant ^15^N transfer within the shoot, tissue homogenized material was weighed (2–4 mg) into 6 × 4 mm^2^ tin capsules (Sercon Ltd.). The samples were analyzed using a continuous flow IRMS (PDZ 2020 IRMS, Sercon Ltd), with air used as the reference standard. Percentage N and atom percentage ^15^N was measured. ^15^N transferred from fungus‐to‐plant was calculated using the following equation:

$$M_{\text{Ex}}=\left(\frac{At_{\text{lab}}-At_{\text{cont}}}{100}\right)\left(M\left[\frac{\%N}{100}\right]\right).$$

where *M*_Ex_ is the isotope mass (excess) (g); *At*_lab_ is the atom percentage of the isotope in labeled microcosm; *At*_cont_ is the atom percentage of the isotope in paired control microcosm, *M* is the sample biomass (g), and %N is the nitrogen percentage (Cameron et al., 2006).

### 2.8 Statistical analysis

The impact of AMF inoculum and wheat cultivar on measured parameters was assessed by two‐way ANOVA followed by Tukey HSD tests using Minitab (Version 17). Before statistical analysis, data were checked for conformation to normality and equal variance assumptions using normal probability plots and residuals versus fits plots. Data which did not conform to assumptions were transformed using the optimal lambda function in Minitab (Version 17). The heteroscedasticity of the concentration and total ^33^P data could not be made to fit ANOVA assumptions through transformation. Therefore, Student's *t* tests were performed between AMF treatments within each wheat cultivar on Minitab (Version 17).

AMF community fingerprint data collected from T‐RFLP analysis was analyzed using the Vegan: Community Ecology Package (R package version 2.5‐6). A permutational multivariate analysis of variance (PERMANOVA) was conducted using the “adonis” function in vegan, to assess whether communities differed significantly between inoculated and uninoculated plants or different wheat cultivars. The homogeneity of group dispersions assumption was assessed by the “betadisper” function, which is a multivariate equivalent of Levene's test for homogeneity of variances. Differences were evaluated visually by boxplots and by ANOVA, and no significant differences in dispersion were detected.

## 3 RESULTS

### 3.1 AMF colonization and community composition changes

**Figure 1:**
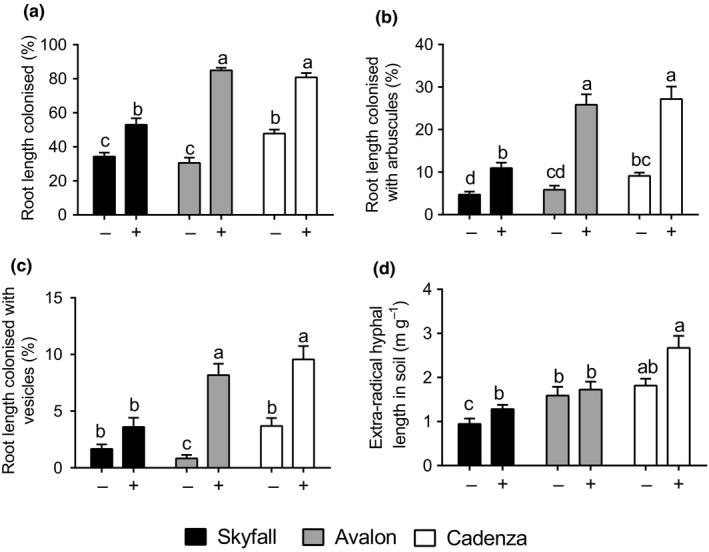
Root colonization by arbuscular mycorrhizal fungi: (a) total colonization, (b) arbuscules, (c) vesicles, and (d) extra‐radical hyphal lengths for three wheat cultivars, Skyfall (black bars), Avalon (grey bars), and Cadenza (white bars). Plants were grown in agricultural, non‐sterile soil inoculated with commercially obtained arbuscular mycorrhizal fungi inoculum containing *Rhizophagus irregularis* (+) or a sterilized control (−). Error bars represent the standard error of the mean. Different letters refer to significant differences (*n* = 12, *p* < .05, two‐way ANOVA, TukeyHSD post‐test) between treatments

Root length colonization by AMF varied between a mean of 31%, 34%, and 48% in cv. Avalon, Skyfall, and Cadenza, respectively, grown in non‐inoculated agricultural soil. Inoculating plants resulted in significantly greater mycorrhizal colonization within the roots (*F* = 257.2, *df *= 1.66, *p* < .001; two‐way ANOVA; Figure 1a). However, the extent of colonization increase varied between wheat cultivars (Interaction: *F* = 22.17, *df *= 2,66, *p* < .001; two‐way ANOVA; Figure 1a). cv. Skyfall displayed the smallest increase in root colonization of the wheat varieties tested (54.4%), followed by cv. Cadenza (68.9%) with cv. Avalon increasing the most (177.9%). AMF structures (arbuscules and vesicles) were more abundant within the root systems of inoculated than non‐inoculated wheat plants (arbuscules: *F* = 136.5, *df *= 1.66, *p* < .001; two‐way ANOVA; Figure 1b; vesicles: *F* = 72.0, *df *= 1.66, *p* < .001; two‐way ANOVA; Figure 1c).

Adding AMF inoculum resulted in significantly increased hyphal density within the soil, when compared to plants grown in agricultural soil alone (*F* = 10.24, *df *= 1.66, *p* < .01; two‐way ANOVA; Figure 1d). There were also differences in soil hyphal lengths between cultivars (*F* = 22.18, *df *= 2.66, *p* < .001; two‐way ANOVA), regardless of inoculation treatment (Tukey: *p* > .05).

Inoculation had a significant impact on community composition (*F* = 60.0, *df *= 1.42, *p* < .001; PERMANOVA), and AMF communities were unchanged by wheat cultivar (*F* = 1.3, *df *= 2.42, *p* > .05; PERMANOVA). The commercially inoculated plants had a substantial increase in T‐RFs at 273 bp. Sequences of *R. irregularis* were downloaded from GenBank and virtually digested with restriction enzymes; HpyCHIV produced a T‐RF of 273 ± 1 bp, suggesting *R. irregularis*, which was present only sporadically in non‐inoculated plants, became much more frequent in roots after *R. irregularis* inoculum was added.

### 3.2 The effect of AMF inoculation on plant nutrient uptake

**Figure 2:**
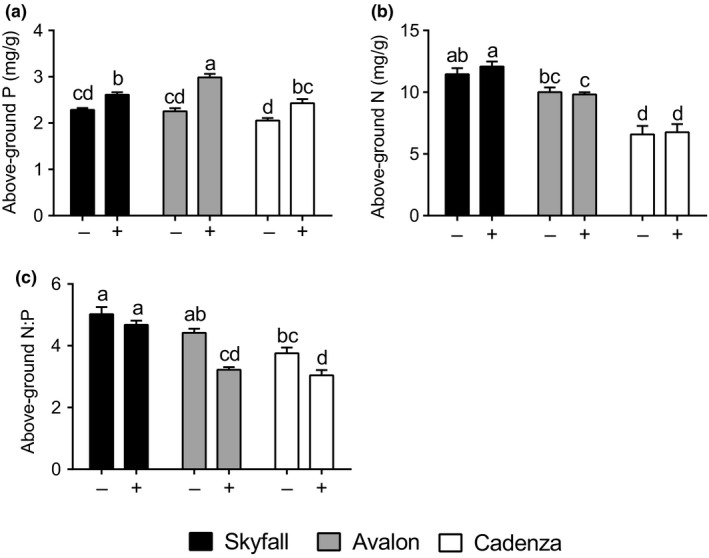
(a) Plant shoot tissue concentration of P, *n* = 12. (b) Plant shoot tissue concentration of N, *n* = 10. (c) N:P of above‐ground plant tissues, *n* = 10. Plants were grown in a non‐sterile agricultural soil with (+) or without (−) active AMF inoculation with *R. irregularis*. Experiments were conducted in three wheat cultivars, Skyfall (black bars), Avalon (grey bars), and cadenza (white bars). Error bars represent the standard error of the mean. Different letters refer to significant differences between treatments. *p* < .05, two‐way ANOVA, TukeyHSD posthoc test

Inoculated plants contained more P in above‐ground tissues than those grown in non‐inoculated agricultural soil in terms of both absolute quantity and concentration. P in the shoots was greater by 14.3%, 32.4%, and 18.2%, in inoculated cv. Skyfall, Avalon, and Cadenza, respectively, than their counterparts grown in agricultural soil alone (Interaction: *F* = 5.54, *df *= 2.66, *p* < .01; two‐way ANOVA; Figure 2a).

Inoculation did not increase N concentrations (*F* = 0.13, *df *= 1.54, *p* = .718; two‐way ANOVA; Figure 2b) or total N within above‐ground plant tissues (*F* = 0.91, *df *= 1.54, *p* = .344; two‐way ANOVA) in any cultivars tested. The increase in P, but not N, in the shoot material when plants were inoculated with *R. irregularis* resulted in lower N:P ratios of plants grown in inoculated compared to non‐inoculated soil (*F* = 31.6, *df *= 1.54, *p* < .001; two‐way ANOVA; Figure 2c).

### 3.3 Bi‐directional exchange of ^33^P and ^15^N for C between wheat and AMF

**Figure 3:**
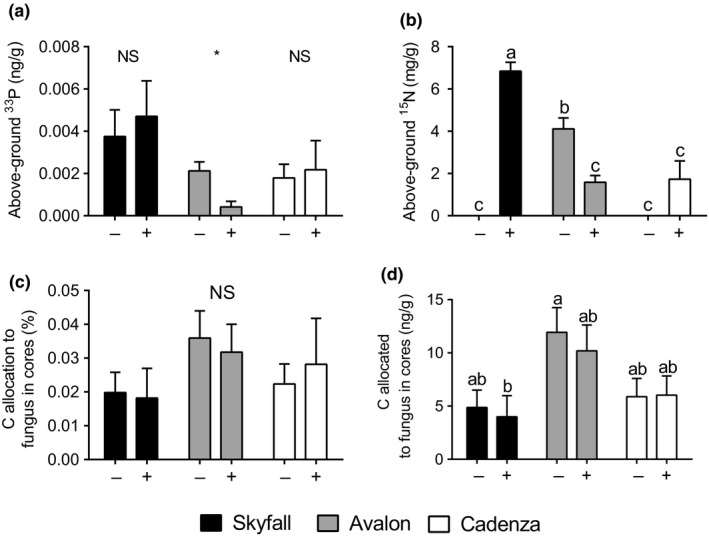
(a) Plant shoot tissue concentration of ^33^P assimilated via fungal symbionts, *n* = 6. (b) The concentration of fungal‐assimilated ^15^N in plant shoot tissues, *n* = 6. (c) Percentage allocation of plant‐derived C to fungi within cores, *n* = 12. (d) Concentration of C allocation to fungus in the cores, *n* = 12. Plants were grown in agricultural, non‐sterile soil inoculated with commercially obtained arbuscular mycorrhizal fungi inoculum containing *Rhizophagus irregularis* (+) or a sterilized control (−). (a) Student's *t* tests, *p* < .05, (b–d) data transformation by optimal lambda to fit the assumptions of ANOVA, *p* < .05, two‐way ANOVA, TukeyHSD posthoc test. Different letters refer to significant differences between treatments. * refers to significant differences between treatments. ns refers to no significant differences

The functionality of the fungal hyphal networks was determined by quantifying the ^33^P and ^15^N in the above‐ground plant tissues in pots where the isotopes were introduced into static cores after values from plant tissue where ^33^P and ^15^N was introduced to rotated cores was subtracted. There was no increase in above‐ground [^33^P] in any wheat cultivars as a result of AMF inoculation (Figure 3a). cv. Avalon assimilated significantly more ^33^P from its fungal partner(s) when grown in non‐inoculated agricultural soil with only the native AMF community present than when the soil was supplemented with commercial *R. irregularis* inoculum (*t* = 3.34, *df *= 8, *p* = .01; Student's *t* test). In contrast, there was no significant difference in ^33^P acquired from AMF partners when cv. Skyfall (*t* = 0.45, *df *= 9, *p = *.66*;* Student's *t* test) and cv. Cadenza (*t* = 0.25, *df *= 7, *p* = .81; Student's *t* test) were inoculated compared to when they were grown in non‐inoculated agricultural soil. Overall, there were cultivar‐specific differences in mycorrhiza‐acquired ^33^P with cv. Skyfall assimilating more ^33^P via AMs than the other wheat cultivars tested.

Adding AMF inoculum to an agricultural soil significantly affected ^15^N assimilation in wheat with differences driven by wheat cultivar (Interaction: *F* = 51.0, *df *= 2.30, *p* < .001; two‐way ANOVA; Figure 3b). In non‐inoculated agricultural soil, cv. Skyfall assimilated no mycorrhiza‐acquired ^15^N. However, when the soil was inoculated, mycorrhiza‐acquired ^15^N increased to 6.8 μg/g, the highest of all treatments. In contrast, there was a significant decrease in mycorrhiza‐acquired ^15^N when cv. Avalon was inoculated, compared to plants grown in agricultural soil only, from 4.1 μg/g to 1.6 μg/g. cv. Cadenza had the lowest mycorrhiza‐acquired ^15^N concentration in the shoot material and was not significantly affected by adding *R. irregularis* inoculum.

Despite there being greater root colonization by AMF when soil was supplemented with the commercial AMF inoculum, C allocated to fungal symbionts was not altered in any of the wheat cultivars (*F* = 1.0, *df *= 1.66, *p* = .35; two‐way ANOVA; Figure 3c,d). However, cultivar‐specific differences were observed in the amount of C allocated to extra‐radical hyphae, with cv. Avalon allocating more than double the amount of C to its fungal partner than cv. Skyfall or cv. Cadenza (*F* = 4.9, *df *= 2.66, *p* = .01; two‐way ANOVA).

### 3.4 Plant biomass

**Figure 4:**
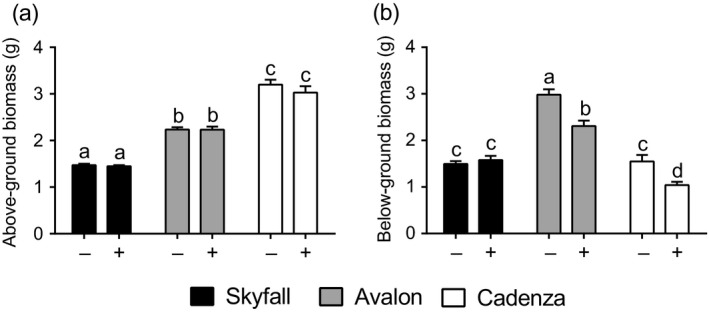
(a) Above‐ and (b) below‐ground plant biomass. Experiments were conducted in three wheat cultivars, Skyfall (black bars), Avalon (grey bars), and Cadenza (white bars). Plants were inoculated with arbuscular mycorrhizal fungi inoculum containing *Rhizophagus irregularis* (+) or a sterilized control (−). Error bars represent the standard error of the mean. Different letters refer to significant differences between treatments (*n* = 12, *p* < .05, two‐way ANOVA, TukeyHSD post‐test, above‐ground tissue biomass data transformed by optimal lambda

The use of AMF inoculum in agricultural soil had no impact on above‐ground biomass in wheat, across all cultivars tested (*F* = 1.15, *df *= 1.66, *p* > .05; two‐way ANOVA; Figure 4a). In two wheat cultivars (Avalon, Cadenza), inoculation with AMF resulted in a smaller root system compared to the control plants, but this was not the case in cv. Skyfall (Interaction: *F* = 7.38, *df *= 2.66, *p* = .001; two‐way ANOVA; Figure 4b).

## 4 DISCUSSION

Supplementing agricultural soil with a commercial AMF inoculant dramatically increased wheat root colonization by AMF and significantly changed the fungal community colonizing the roots. While soil processing (sieving) in preparation for the experiment may have had an additional disturbance effect, our findings strengthen evidence that the inoculum potential of agricultural soil has become impaired by conventional farming practices (Lekberg & Koide, 2005) which reduce AMF propagule abundance and diversity (Jansa et al., 2002; Bowles, Jackson, Loeher, & Cavagnaro, 2017). Although increased root colonization following inoculation with AMF products has previously been recorded (Lekberg & Koide, 2005; Köhl, Lukasiewicz, & van der Heijden, 2016), this is the first evidence that inoculation with commercial *R. irregularis* inoculum has a similar effect across several wheat cultivars. However, in order for mycorrhizal inoculation to be considered “successful,” and form part of a sustainable food production system, it must impart a measurable benefit on the host plant(s), such as increased nutrient assimilation.

Few investigations have focused on effectiveness of mycorrhizal inocula in increasing mycorrhiza‐acquired nutrients in the target plants. We found adding AMF inoculum not only resulted in higher root colonization but also enhanced the total P content of above‐ground plant tissues across all wheat cultivars tested. Substantially higher plant P uptake of up to 32% in inoculated wheat, plus evidence of higher plant P concentrations in field studies of inoculated wheat (Mohammad, Mitra, & Khan, 2004), suggests that there is potential for AMF inoculants to reduce demand for P fertilizers in agricultural systems. However, AMs are rarely responsible for the entirety of plant P assimilation; plants engage in their own direct P uptake via the root epidermis and root hairs, in addition to the indirect mycorrhizal P pathway (Smith et al., 2011). We quantified ^33^P assimilation via the mycorrhizal pathway in wheat, grown in non‐sterile agricultural soil, inoculated with AMF or sterilized carrier only. We found no increase in the amount of mycorrhiza‐acquired ^33^P in plants inoculated with active AMF compared to those inoculated with the sterilized carrier. Thus, despite greater total P in plant tissues of AMF‐inoculated plants, it is not directly attributable to the increased root colonization by AMF. While this finding seems counterintuitive, it is important to note that total P quantification represents P accumulation over the lifetime of the plant, whereas ^33^P uptake was measured only for the last ten days of the experiment. It is possible that AMs contribute most to plant P assimilation in the earlier stages of plant development, which were not measured during this experiment (van der Heijden, 2004; Kobae, 2019). However, given that our isotope tracing experiments were conducted during shoot elongation and rapid growth, the direct contributions of AMs to wheat P assimilation remain unclear.

AM symbiosis can alter a plant's physiology and environment in ways which may enhance nutrient uptake, without direct P contribution through the fungal hyphae. It has been shown that plant P transporter gene expression can change in response to AMF colonization (Paszkowski, Kroken, Roux, & Briggs, 2002; Glassop, Smith, & Smith, 2005), potentially altering plant P uptake, although changes in P transporter expression and changes in P uptake have seldom been linked (Grønlund et al., 2013). Additionally, AMF have a profound effect on the wider soil microbial community, as a sink for plant photosynthates (Johnson, Leake, Ostle, Ineson, & Read, 2002) they provide a soil C source through hyphal turnover and energy‐rich exudates (Zhang, Lehmann, Zheng, You, & Rillig, 2018). This C supply may stimulate soil bacteria, including those shown to increase plant P uptake such as P solubilizing bacteria (Toljander, Lindahl, Paul, Elfstrand, & Finlay, 2007; Zhang et al., 2018). Given that we used non‐sterilized agricultural field soil, it is likely a combination of these factors resulted in the greater tissue P accumulation in inoculated plants. Additionally, there is evidence that non‐sterile soil can suppress extra‐radical hyphal development and P uptake through a combination of abiotic and biotic factors, such as certain bacteria and fungi, which can be mitigated by pasteurization and to a lesser extent liming to increase the soil pH (Svenningsen et al., 2018; Cruz‐Paredes et al., 2019). Suppression by certain soil bacterial and fungal species could partially explain why mycorrhiza‐acquired ^33^P uptake by the plants represented a small proportion of ^33^P added to the cores in our experiments. Alternatively, the wheat cultivars tested may simply be relatively unresponsive to AMs due to generations of commercial breeding selecting for root systems that are effective at assimilating nutrients from mineral fertilizers (Tawaraya, 2003), leading to modern crop cultivars that are less reliant on AMs (Hetrick, Wilson, & Cox, 1993; Zhu, Smith, Barritt, & Smith, 2001).

Our data provide no evidence that AMF inoculation could increase N uptake in wheat hosts, although there appears to be a small amount of AM transfer of ^15^N to host plants (Govindarajulu et al., 2005; Jin et al., 2005). Our results are in line with the long‐held view that while plant hosts may assimilate N via AMs, it is quantitatively insignificant in comparison to P acquisition. AMF may be more beneficial for plant P uptake because inorganic N sources are more mobile in the soil and consequently do not become depleted in the rhizosphere limiting root access (Smith & Smith, 2011). Notably, many studies which show an improvement in AMF‐associated N assimilation have not analyzed the root system fungal community. Evidence is emerging that Mucoromycotina fine‐root endophytic (MFRE) fungi are widely associated with land plants and often found in dual association with Glomeromycotina fungi (Field et al., 2019; Hoysted et al., 2019). Mucoromycotina fine‐root endophytic (MFRE) fungi may be important for N assimilation, and co‐exist with AMF due to the functional complementarity of their nutritional symbioses (Field et al., 2019; Hoysted et al., 2019).

Even though there were no gains in overall N concentrations in plant tissue in our experiments, the amount of mycorrhiza‐acquired ^15^N varied considerably depending on inoculation and wheat cultivar. For example, cv. Skyfall ^15^N uptake increased substantially when inoculated with *R. irregularis*, potentially due to community changes in the roots or to the rhizosphere community around the roots to more favorable symbiotic partners. In contrast, when cv. Avalon was inoculated with *R. irregularis* mycorrhiza‐acquired ^15^N decreased significantly, suggestive of cultivar specificity in AMF symbiotic function. AMF community composition did not differ between the cultivars after inoculation, and a T‐RF likely representing *R. irregularis* dominated the root community. Therefore, the cultivar differences may be due to a cultivar‐specific compatibility with the *R. irregularis* isolate used in this experiment, with cv. Avalon responding negatively with both ^33^P and ^15^N assimilation lower after inoculation. The cultivar differences demonstrated in this experiment reinforce the importance of adequate testing before inoculant implementation in the field. Inoculants will not be beneficial in all cases and synergistic consortia of AMF tailored to different crops and environments may be needed.

Plants often allocate large amounts of photosynthate to their fungal partners (Johnson, Graham, & Smith, 1997), and this “drain” on C resources could be responsible for negative growth responses to AMs (Graham & Abbott, 2000; Li, Smith, Dickson, Holloway, & Smith, 2008). In our experiments, wheat did not allocate substantial amounts of photosynthetically fixed C to fungal symbionts across all cultivars tested. When C allocated to fungi within the static core was scaled up to the entire pot, the percentage of plant‐fixed C allocated to AMF was negligible and wheat cultivar dependent, with the highest being in cv. Avalon at 2%. The amounts of ERM in the soil also differed between wheat cultivars but could not be explained by the wheat cultivar‐dependent C allocation to the fungi. C allocation was lower than general estimates that between 4% and 20% of photosynthate is allocated by plant hosts to fungal partners (Smith & Read, 2008), again suggesting that AMF functional compatibility has been all but lost from modern wheat cultivars, potentially via selection for above‐ground traits. However, recent evidence suggests plant carbon allocation to AMF may be lower than previously estimated and frequently falls below 10% of the plant C budget (Konvalinková, Püschel, Řezáčová, Gryndlerová, & Jansa, 2017).

Despite finding near‐negligible plant C allocation and a higher P uptake (through AMs, roots or alternative microbial cycling processes) with inoculation, we recorded no increase in above‐ or below‐ground biomass. One possibility for the lack of growth response, despite increases in tissue P, is that the plants were N, rather than P, limited. This is supported by low N:P and low N concentrations in plant tissues. Therefore, AMF inoculants may have potential to improve plant biomass in P limited conditions, which could be common in future agricultural systems. Unfortunately, plants in our experiments were not grown to yield due to the necessity of conducting isotope tracing during a period of rapid growth and nutrient uptake. Therefore, future research should have a strong focus on the yield benefits commercial inoculants could achieve under these conditions, including the nutritional quality of the grain which is a key parameter of food security (Myers et al., 2014). A recent meta‐analysis shows AMF inoculants improved yields in many crop varieties, including wheat, by an average of 17% (Zhang et al., 2018). Uptake of micronutrients such as zinc, copper, and iron can also be enhanced by AMs (Lehmann, Veresoglou, Leifheit, & Rillig, 2014; Lehmann & Rillig, 2015) and could have important implications for crop production and human health.

Our results show that the AMF inoculum tested has potential to form a useful component of a sustainable agricultural management system, to boost wheat root colonization by AMF and reduce crop dependence on P‐fertilizer inputs, the raw materials for which are a vital but non‐renewable resource (Cordell et al., 2009). Cultivar differences in AM‐acquired nutrients demonstrate that genetic variation within wheat germplasm may determine AM‐acquired benefits, with many studies reporting significant differences among crop varieties in their response to mycorrhizal associations, suggesting breeding programs could capitalize on these genetic differences (Fester & Sawers, 2011). The potential genetic basis for AMF colonization (Lehnert, Serfling, Enders, Friedt, & Ordon, 2017) and AM effects on drought tolerance in wheat (Lehnert, Serfling, Friedt, & Ordon, 2018) have recently been reported which, together with our findings and future research in the area, could form the basis of such breeding programs. The inclusion of markers for AMF responsiveness (i.e., colonization potential, response to abiotic change, and nutritional function) in these programs will lead to development of new crop varieties which could be successfully combined with commercial inoculum, thereby reducing the risk of P limitation and going some way to reducing agricultural reliance on chemical fertilizers.

## AUTHORS’ CONTRIBUTIONS

A.J.E., D.D.C., T.J.D., and K.J.F. conceived and designed the research. A.J.E. conducted experimental work and analyses. A.J.E., T.J.D., and K.J.F. discussed the results and A.J.E. led the writing. All authors edited and provided comments on the manuscript. We thank the three anonymous reviewers and the editor for their constructive comments on our manuscript.
