# Supplementary material for: A commercial arbuscular mycorrhizal inoculum increases root colonization across wheat cultivars but does not increase assimilation of mycorrhiza‐acquired nutrients
Source: Plants People Planet. 2020 Jan 3;3(5):588–99. doi: 10.1002/ppp3.10094 (PMC8607474; doi:10.1002/ppp3.10094)
Supplement: Supplementary file 1 [file PPP3-3-588-s001.pdf]

Plants, People, Planet **Supporting Information**

Article title: Commercial arbuscular mycorrhizal inoculum increases root colonisation in wheat but does not increase assimilation of mycorrhizal-acquired nutrients

Authors: Ashleigh J. Elliott[1], Tim J. Daniell[2], Duncan D. Cameron[2] and Katie J. Field[1]*

The following Supporting Information is available for this article:

Fig. S1 Schematic diagram of mesh-covered core (not drawn to scale).

Fig. S2 Experimental systems for tracing ^{33}P and ^{15}N-for-^{14}C exchange between mycorrhizal fungi and plant partners.

Methods S1 This file provides specific details on methods referred to in the main text, including biological materials and growth conditions, equations for calculation of phosphorus and carbon exchange between symbionts. Additional methods for measuring extraradical hyphal lengths, PCR conditions, restriction enzyme digests and Genemapper analysis.

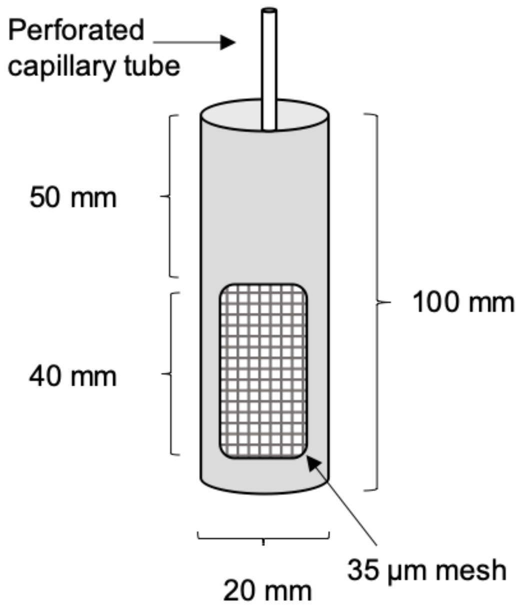

Figure S1: Schematic diagram of mesh-covered core (not drawn to scale).

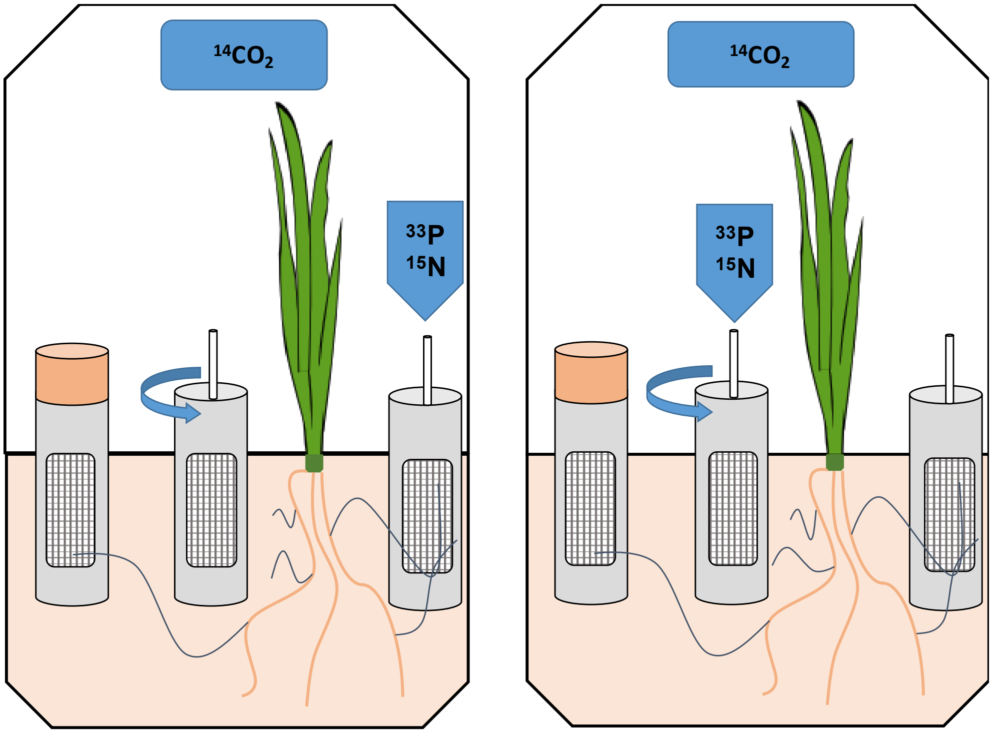

Figure S2: Experimental systems for tracing ^{33}P and ^{15}N-for-^{14}C exchange between mycorrhizal fungi and plant partners. Mesh covered cores were labelled with ^{15}N-ammonium chloride and ^{33}P-orthophosphate which only the intact fungal hyphae could access in the static core treatment. In the rotated control treatment, the labelled core was rotated to sever hyphal connections and control for isotope diffusion outside of the core. $^{14}CO_2$ was released into sealed chamber to measure the fixation of C by the wheat plants and transfer to fungi within the static cores. The third core with an airtight seal (orange) was used to track plant-fixed carbon passed to the fungal partner through fungal respiration of ^{14}C.

Materials and Methods

Biological material and growth conditions

Wheat seedling were sterilised with chlorine gas, germinated on damp filter paper at 20°C for six days then transplanted into pots containing agricultural soil and sand in a 1:1 mix. Agricultural soil was collected from Leeds University Farm (Spen Common Lane, Tadcaster, North Yorkshire, LS24 9NU, England), sand was added to reduce compaction to aid water drainage. The soil, a slightly alkaline sandy clay loam with a pH of 7.5, was air-dried and passed through a 2 mm sieve. Analysis of the soil characteristics showed soil organic C content represented ~2% of soil dry weight and soil solute concentrations of PO_4, NO_3, and NH_4 were 0.08 mg L^{-1}, 6 mg L^{-1}, and 0.04 mg L^{-1} respectively (Holden et al., 2019). At the time of planting, the wheat seedlings were inoculated with a commercially available inoculum (PlantWorks, Kent, UK), which contained a mixture of infective AMF propagules including colonised root fragments, hyphae and spores (100 per gram) and an inert carrying substrate without fertiliser additions (1:1 pumice and zeolite). The recommended application rate for the inoculum is ~ 6 g per 1 L pot.

At the time of planting, mesh windowed cores were inserted into the pots perforated capillary tubes were fixed inside the cores using a waterproof silicon sealant (Aqua Mate, Ever Build, Dublin, Ireland) applied to the end of the tube before it was placed onto the mesh at the bottom of the core. The cores were filled with the agricultural soil/sand growing substrate contained in the rest of the pot. Greenhouse growing conditions were supplemented with LED lighting and electronic blinds to create a 16-hour photoperiod, light intensity: 350 μmol.m^{-2} s^{-1}, average temperature: 23°C, the plants were watered every three days with each being given 30 ml of 40% nitrate type Long Ashton solution weekly (Hewitt, 1966).

Quantifying ^{33}P- and ^{15}N-for-C exchange between wheat and fungi.

Below-ground respiration was sampled every 2 hrs and above-ground gas throughout the labelling period by injecting gas samples into gas-evacuated scintillation vials containing 10 ml of the C trapping chemical CarbonTrap (Meridan Biotechnologies) and mixed with 10 ml scintillation chemical CarbonCount (Meridan Biotechnologies). Sample activity was monitored by scintillation

counting (Packard Tri-carb 3100TR, Isotech, Chesterfield, UK). At the end of the 16h photoperiod, 4 ml 2M KOH was injected into vials within the sealed systems to trap remaining $^{14}CO_2$ gas.

Mycorrhizal-acquired ^{33}P

To quantify the amount of ^{33}P transferred from fungus to plant, samples (30-50 mg plant or 40-100 mg soil) were digested in 1 ml concentrated sulphuric acid at 365°C for 15 minutes. Upon cooling, 100 µl hydrogen peroxide (Acros Organics, Geel, Belgium) was added to the samples and reheated to 365°C for 1 minute and repeated until the solution cleared. The cleared sample was diluted up to 10 ml with distilled water. ^{33}P activity was quantified via Packard Tri-carb 3100TR (Isotech, Chesterfield, UK) using 2 ml of digest solution with 10 ml Emulsify-safe (Perkin-Elmer). ^{33}P transfer between symbionts was corrected for radioactive decay and measured using Equation 1 in Cameron et al., 2007 .

$$M_{33P} = \left\{ \left[\frac{(C_{DPM}/60)}{S_{Act}} \right] M_{wt} \right\} D_f$$

Equation 1: *Where M_{33P} = mass of ^{33}P (mg), C_{DPM} = counts as disintegrations per minute, S_{Act} = specific activity of the source (Bq mmol^{-1}), D_f = dilution factor (in this case 10) and M_{wt} = molecular mass (of P) (Taken from Cameron et al. 2007).*

Transfer of carbon from plant to fungus

Between 10 and 40 mg of plant tissue or soil was placed in a Combusto-cone (Perkin Elmer) and the CO_2 released through oxidation was trapped in 10 ml CarbonTrap (Meridan Biotechnologies) and mixed with 10 ml CarbonCount (Meridan Biotechnologies). Radioactivity present in the sample measured using the following equations.

$$M_{14_c} = \left(\frac{\left[\frac{C_{DPM}/60}{10^9} \right]}{SP_{Act}} \right) M_{wt}$$

***Equation 2**: (M$_{14c}$, the mass of ^{14}C (mg); C_{DPM}, counts as disintegrations per minute; SP_{Act}, the specific activity of the source (Bq mmol^{-1}); M_{wt}, molecular mass (of C)* (Cameron et al., 2006)*).*

$$T_{pf} = \left(\left(\frac{A}{A_{sp}}\right)m_a\right) + (P_r \times m_c)$$

Equation 3: *(T_{pf}, total carbon transferred from plant to fungus in any given pool (g); A, radioactivity (Bq); A_{sp}, specific activity of the source (Bq Mol^{-1}); and m_a, atomic mass of the element, in this case the isotope ^{14}C; P_r, proportion of the total ^{14}C label supplied (as $^{14}CO_2$) that is present in the tissue; m_c, mass of C in the CO_2 present in the labelling chamber in m^3 (from the ideal gas law, Eqn 4) (Taken f*rom Cameron et al., 2008)

$$m_{cd} = M_{cd}\left(\frac{PV_{cd}}{RT}\right) thus\ m_c = m_{cd} \times 0.27292$$

Equation 4: *(m_{cd}, mass of CO_2; M_{cd}, molecular mass of CO_2; P, pressure; V_{cd}, volume of CO_2 in the chamber (V_{cd} = 0.044% of the total chamber volume based on an atmospheric [CO_2] of 440 ppm or 800 ppm); m, mass of unlabelled C in the labelling chamber; M, molar mass (M of C = 12.011 g); R, universal gas constant; T, absolute temperature; m_c, mass of C in the CO_2 present in the labelling chamber in m^3; 0.27292 represents the proportion of C in CO_2 on a mass fraction basis (Taken from Cameron et al. 2008)).*

Hyphal extractions and measuring extraradical hyphal lengths

Hyphal lengths were measured by a grid-line-intersect method over 50 fields of view (Tennant, 1975). A 1-3 g sample of soil was dispersed in 1000 mL of water on a stirring plate and hyphae were extracted from the spinning liquid via a syringe. This liquid was then dispensed into the Millipore filtration apparatus attached to a vacuum pump. The fluid was drawn through a filter paper disc which caught the hyphae, which was subsequently dyed with a trypan blue stain.

PCR and T-RFLP

The PCR was carried out in a 20 μl reaction using 2 μl of DNA template, 10 μl of Qiagen Mastermix (Qiagen, Hilden, Germany) and 0.5 μM of each primer, made up to the final volume with PCR water. Thermal cycling consisted of an initial DNA denaturation step of 3 min at 94°C followed by 35 cycles each of 30 s at 94°C, 40 s at 59°C and 60 s at 72°C with a final extension step of 10 min at 72°C, on a 96-well thermal cycler. All PCR plates included a negative control to ensure

that no DNA contamination was present. Gel electrophoresis was used to verify the success of PCR amplification. 1 µl of loading buffer (Bioline, London, UK) and 4 µl of PCR product was run on a 1.5% agarose gel with 0.001 % (v/v) SYBR® Safe DNA stain (Invitrogen, Carlsbad, USA). PCR products were run along-side a 100 bp ladder (Bioline, London, UK).

For the HpyCHIV/MboII/Sau96I digest, the optimal reaction set-up contained 1 unit of each enzyme, 1 µl of CutSmart buffer (All enzymes had 100% activity in CutSmart buffer) and 3 µl of PCR product, made up to final reaction volume of 10 µl with water. Digests were incubated for 60 minutes at 37°C then denatured at 65°C for 20 minutes. All digests were run alongside uncut samples and negative controls. Digests were diluted 1:10 with water to prepare the samples for capillary DNA analysis. 1 µl of the diluted digest was added to 9 µl of formamide containing 1% GeneScanTM LIZ 1200 size standard (Applied Biosystems, UK) and heated at 94°C for 3 minutes before immediate cooling on ice. Genotyping was carried out on an ABI 3730 PRISM® capillary DNA analyser (Applied Biosystems, UK).

T-RFLP data was analysed using Genemapper software v. 5 (Applied Biosystems, UK) with a background threshold of 50 fluorescent units, and a bin width of 5 bp. Peaks were analysed in the range of 50-850 bp. The relative abundance of each peak was calculated to the percentage of total sample fluorescence. Peaks containing <1% of the total sample fluorescence were discarded, and artefacts were detected by identifying peaks which frequently occurred in both the cut and uncut samples, after their removal the proportion of total sample fluorescence accounted for by the peaks was recalculated.

References

Cameron, D. D., Johnson, I., Leake, J. R., & Read, D. J. (2007). Mycorrhizal acquisition of inorganic phosphorus by the green-leaved terrestrial orchid *Goodyera repens*. Annals of Botany, 99(5), 831–834. https://doi.org/10.1093/aob/mcm018

Cameron, D. D., Johnson, I., Read, D. J., & Leake, J. R. (2008). Giving and receiving: Measuring the carbon cost of mycorrhizas in the green orchid, *Goodyera repens*. New Phytologist, 180(1), 176–184. https://doi.org/10.1111/j.1469-8137.2008.02533.x

Cameron, D. D., Leake, J. R., & Read, D. J. (2006). Mutualistic mycorrhiza in orchids: Evidence from plant-fungus carbon and nitrogen transfers in the green-leaved terrestrial orchid *Goodyera repens*. New Phytologist, 171(2), 405–416. https://doi.org/10.1111/j.1469-8137.2006.01767.x

Holden, J., Grayson R. P., Berdeni, D., Bird, S., Chapman, P. J., Edmondson, J. L., Firbank, L. G., Helgason, T., Hodson, M. E., Hunt, S. F. P., *et al.* (2019). The role of hedgerows in soil functioning within agricultural landscapes. Agriculture, Ecosystems and Environment, 273, 1–12. https://doi.org/10.1016/j.agee.2018.11.027

Tennant, D. (1975). A test of a modified line intersect method of estimating root length. Journal of Ecology, 63(3), 995–1001. Retrieved from http://www.jstor.org/stable/2258617
